# Primary hydatid cyst in the adductor muscles of thigh: A case report

**DOI:** 10.1002/ccr3.6664

**Published:** 2022-12-05

**Authors:** Mahnaz Arian, Marzieh Kazerani

**Affiliations:** ^1^ Department of Infectious Diseases, Faculty of Medicine Mashhad University of Medical Sciences Mashhad Iran; ^2^ Department of Infectious Disease Mashhad Islamic Azad University of Medical Sciences Mashhad Iran

**Keywords:** echinococcosis, hydatid cyst, intramuscular, muscular, soft tissue

## Abstract

Hydatidosis rarely (2%–3% of all cases) manifests as an intramuscular mass in endemic areas. This 55‐year‐old farmer with an asymptomatic thigh mass from 35 years ago presented with exertional pain in the last 40 days of the disease course. The patient started on albendazole and was cured with surgery.

## 1 INTRODUCTION

Cystic echinococcosis (hydatidosis) is caused by *Echinococcus granulosus*, a tapeworm from the Taeniidae family. It rarely manifests as an intramuscular mass in endemic areas (2%–3% of all cases).^1^ Herein we report a case of muscular hydatidosis, which presented as an asymptomatic mass for 35 years.

## 2 CASE PRESENTATION

**FIGURE 1:**
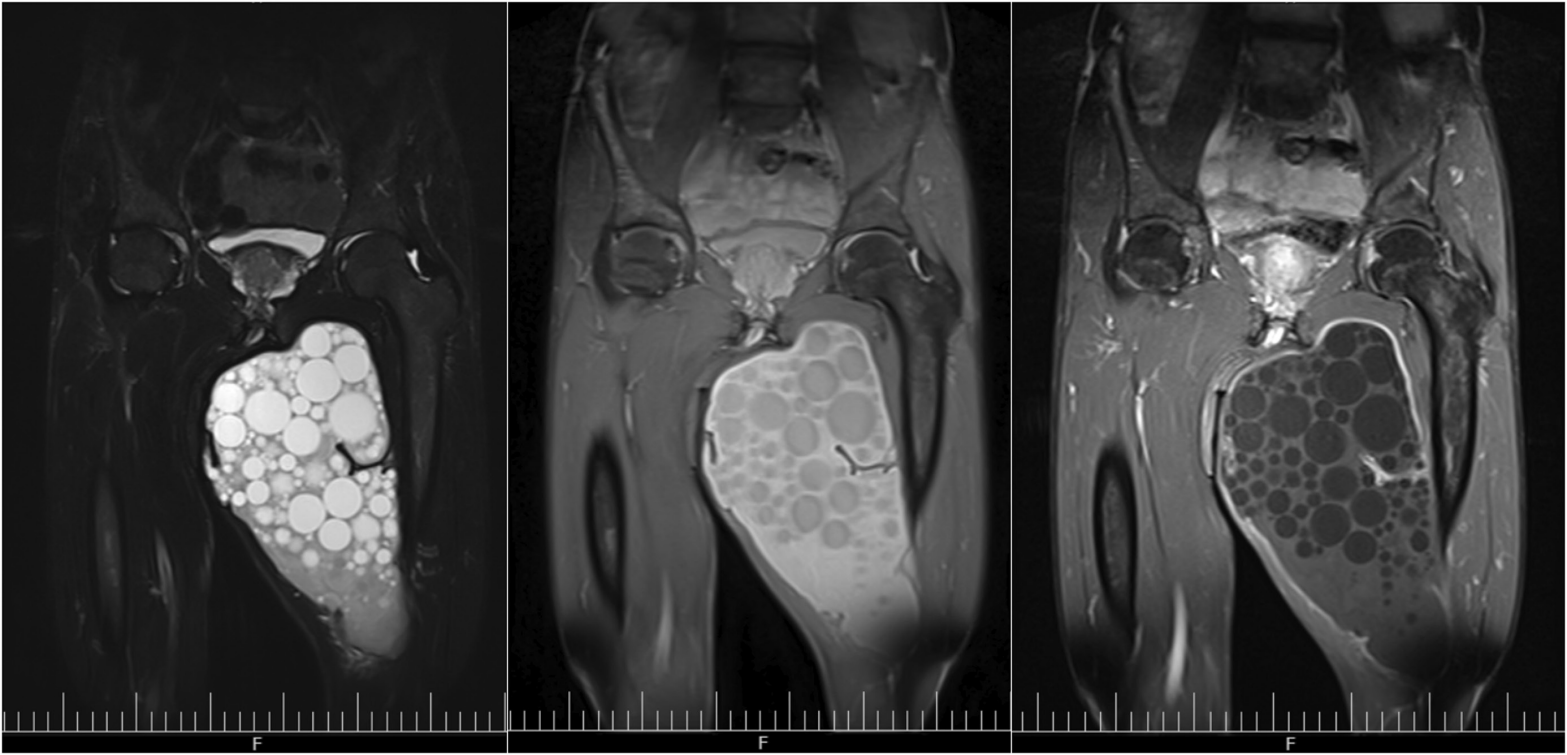
MRI findings of the cyst show numerous daughter cysts in a lobulated cyst in the left thigh

**FIGURE 2:**
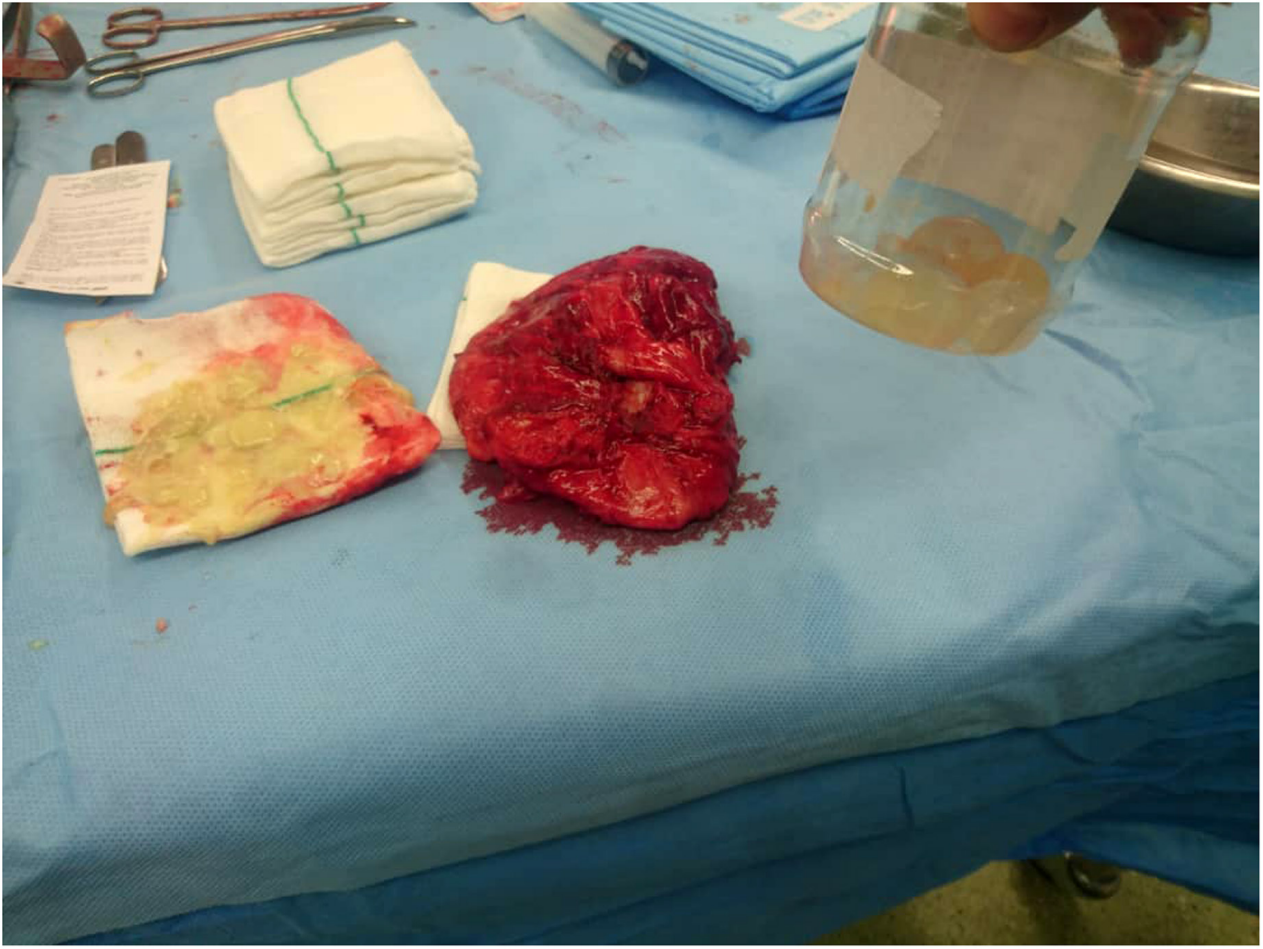
Macroscopic features of the mass

A previously healthy, 55‐year‐old farmer was presented with left thigh pain and swelling that started 40 days earlier. It was aggravated by walking, but no history of fever. He affirms that the mass existed for about 35 years and was not interacting with his daily activities. He used no medication but regularly smoked opium. General examination was unremarkable except for his left thigh. A soft, fluctuating, non‐tender, non‐erythematous mass was palpated on the medial side of his left thigh, with an approximate size of 10 × 8 cm. Hip range of motion was normal. Vital signs were within normal ranges with no fever. WBC: 9100/mm^3^ (lymph: 29%, PMN: 67%), hemoglobin: 11.7 mg/dl, HCT: 34.1%, platelets: 442/mm^3^, AST: 15, ALT: 17, LDH: 293, ESR: 35/h, and CRP: negative. Ultrasound revealed a hypo‐echoic mass of 100 × 270 mm‐size, containing several cystic lesions with poor vascularity on the proximal medial side of his thigh. MRI showed a multi‐loculated, multi‐cystic mass with lobulated margins within the sheath of the thigh's medial muscle compartment (Figure 1). The reported size was 150 × 180 × 220 mm, suggestive of muscle hydatid cyst or lymphangiectasia. The chest x‐ray was normal. The abdominal ultrasound was also normal. CT scan was performed to find other probable cysts throughout the body, and it found no other involvement of the soft tissue or the bone. Pre‐treatment with albendazole was initiated three days before surgery. Surgery was performed, and a multi‐loculated cyst was observed with calcifications and adhesions to the insertion of the thigh's adductor muscles. Macroscopic (Figure 2) and subsequent microscopic evaluation of the cyst confirmed hydatid disease. Albendazole treatment (400 mg per day) continued for two weeks, and the patient remained asymptomatic after a twelve‐month follow‐up.

## 3 DISCUSSION

Echinococcosis is caused by the genus *Echinococcus* (a member of the Taeniidae family). Two genus members are *E. granulosus* (cause hydatid disease) and *E. multilocularis* (cause alveolar echinococcosis). There are two less important members, *E. vogeli* and *E. oligarthrus* (cause polycystic and uni‐cystic echinococcosis, respectively).^2^ Cystic echinococcosis is seen globally, but the Mediterranean area, Middle East, South America, and East Asia are considered highly endemic regions.^3^, ^4^

Canidae family (dogs, wolves, jackals, foxes) are definite hosts that carry the worm's adult form. Humans, cattle, sheep, horses, and camels are accidental hosts that carry the encapsulated larva and oncosphere.^5^ Hatched eggs in the gastrointestinal tract release oncospheres that penetrate the intestinal wall and enter blood circulation.^3^ The most affected sites are the liver (50%–75%) and lungs (20%–30%).^3^, ^4^ Other organs might be involved (1%–5%), such as the spleen, bone, and soft tissues.^4^ While the primary form is exceedingly rare, soft tissue involvement usually is secondary to another source in the body.^4^, ^5^ The usual sites for muscular hydatid disease are the neck, trunk, hip, thigh, and arms.^4^, ^5^, ^6^ This might be due to less activity and rich vascularization of these areas.^3^, ^4^, ^7^ Cysts in these regions usually remained asymptomatic for several weeks or even years (slow‐growing nature).^5^ Early signs are caused by nerve compression or inflammation of the cyst. Fahmi H. et al. declared that about 70% of the cysts exist on the left side of the body, and only 4% of the thigh hydatidosis accompanies another source of infection.^5^ Laboratory results are usually unremarkable, and eosinophilia might not exist if the cyst remains intact.^3^ Diagnosis is established by imaging and serologic tests (ELISA, Western blot, indirect hemagglutinin antibody).^3^ Laboratory results are usually used as a supportive tool and do not rule out the suspected disease.^3^ When hydatidosis is suspected, imaging is essential to avoid aspiration or biopsy, leading to the infection's seeding in other structures.^1^ Ultrasound is available and cost‐effective. It might detect double‐line signs and daughter cysts. 71% of soft tissue hydatidosis has daughter cysts inside. CT scan not only provides information about daughter cysts and detached membranes but it also assesses bone involvement (bone involvement should be considered when soft tissue hydatidosis exists, and it might not have a thick rim as we see in intrahepatic cysts).^6^ MRI shows the periphery of the mass and cysts present as a multilocular cyst with or without rim sign (a low‐intensity rim around the lesion), and they are mostly high signal intensity on T2 and low signal intensity on T1 weighted images.^5^, ^7^ Treatment options are medical therapy, surgical therapy, pre‐cutaneous aspiration‐injection‐reaspiration (PAIR), and wait‐and‐see management. Definite treatment is the surgical removal of the cyst. Post removal washing of the field with hypertonic saline is recommended.^1^, ^3^ Pretreatment with albendazole is offered to decrease the chances of recurrence and anaphylactic shock during surgery.^6^ When mass location makes surgery impossible, PAIR is used with or without medical therapy.^3^, ^6^

## 4 CONCLUSION

Surgeons are advised to consider pretreatment with albendazole to minimize anaphylactic shock and recurrence. Furthermore, physicians should consider the soft tissue's hydatid disease while approaching a soft tissue mass in endemic areas or a patient with a history of traveling to these areas.

## AUTHOR CONTRIBUTIONS

M. Arian: Encountered the case, wrote manuscript, approved final version. M. Kazerani: Consultant, wrote manuscript, approved final version.

## FUNDING STATEMENT

Self‐fund.

## CONFLICT OF INTEREST

None.

## CONSENT

written and verbal consent was taken from the patient for participating in the study and publication of the results anonymously.

## Data Availability

All data regarding the present study are available by contacting the corresponding author.
